# Assessing the Impact of Vitamin D Supplementation on Respiratory Infections in Children and Adolescents: A Cross-Sectional Study

**DOI:** 10.3390/nu16223953

**Published:** 2024-11-19

**Authors:** Elena Tanase, Larisa Mihaela Marusca, Florin George Horhat, Monica Susan, Razvan Susan, Razvan Horhat, Stefania Dinu, Tiberiu-Liviu Dragomir, Sonia Tanasescu

**Affiliations:** 1 Doctoral School, “Victor Babes” University of Medicine and Pharmacy Timisoara, 300041 Timisoara, Romania; tanase.elena@umft.ro; 2 Laboratory Medicine, “Louis Turcanu” Emergency Hospital for Children, 300011 Timisoara, Romania; 3 Multidisciplinary Research Center on Antimicrobial Resistance (MULTI-REZ), Microbiology Department, “Victor Babes” University of Medicine and Pharmacy Timisoara, 300041 Timisoara, Romania; horhat.florin@umft.ro; 4 Department of Internal Medicine I, Centre for Preventive Medicine, “Victor Babes” University of Medicine and Pharmacy Timisoara, 300041 Timisoara, Romania; susan.monica@umft.ro; 5 Department of Family Medicine, Centre for Preventive Medicine, “Victor Babes” University of Medicine and Pharmacy Timisoara, 300041 Timisoara, Romania; razvansusan@umft.ro; 6 Department of Restorative Dentistry, Faculty of Dentistry, Digital and Advanced Technique for Endodontic, Restorative and Prosthetic Treatment Research Center (TADERP), “Victor Babes” University of Medicine and Pharmacy Timisoara, 300041 Timisoara, Romania; horhat.razvan@umft.ro; 7 Department of Pedodontics, Faculty of Dental Medicine, “Victor Babes” University of Medicine and Pharmacy Timisoara, 300041 Timisoara, Romania; dinu.stefania@umft.ro; 8 Department V of Internal Medicine I and Medical Semiology II, ‘’Victor Babes’’ University of Medicine and Pharmacy Timisoara, 300041 Timisoara, Romania; dragomir.tiberiu@umft.ro; 9 Department of Pediatrics, “Victor Babes” University of Medicine and Pharmacy Timisoara, 300041 Timisoara, Romania; tanasescu.sonia@umft.ro

**Keywords:** vitamin D, respiratory infections, food supplements

## Abstract

Background and Objectives: Recent studies suggest that vitamin D supplementation and higher serum 25-hydroxyvitamin D (25-OHD) concentrations may reduce the incidence of respiratory infections in children and adolescents. This cross-sectional study aimed to evaluate the association between different concentrations of vitamin D supplementation, serum 25-OHD concentrations, and the frequency of respiratory infections among individuals aged 1 to 18 years, for a duration of 2 years. Methods: Concerning sun exposure in relation to vitamin D, the study took place in Romania, at approximately 45-degree northern latitude. A total of 194 patients were divided into groups based on weekly vitamin D supplementation (<400 IU, 400–800 IU, >800 IU), serum 25-OHD concentrations (<20 ng/mL, 20–30 ng/mL, >30 ng/mL), and age (<6 years, 6–12 years, 12–18 years). The overall incidence of respiratory infections was 41.2%. Results: Participants receiving >800 IU/week had a significantly lower incidence of infections (16.7%) compared to those receiving <400 IU/week (60.0%, *p* < 0.001). Similarly, participants with serum 25-OHD concentrations >30 ng/mL had an infection rate of 16.7%, compared to 61.4% in those with concentrations <20 ng/mL (*p* < 0.001). Age-specific analyses revealed that the protective effect of vitamin D was most pronounced in children under 6 years old. Logistic regression showed that higher vitamin D supplementation and serum 25-OHD concentrations were independently associated with reduced odds of respiratory infections (OR = 0.25 and OR = 0.22, respectively, *p* < 0.001). Conclusions: These findings support the potential role of vitamin D supplementation in preventing respiratory infections in the pediatric population.

## 1. Introduction

Vitamin D, a fat-soluble vitamin, plays a crucial role in calcium homeostasis and bone metabolism [1,2]. It is synthesized in the skin upon exposure to ultraviolet B (UVB) radiation and obtained from dietary sources, including fatty fish and fortified foods [3]. Beyond its classical functions, vitamin D has been recognized for its immunomodulatory effects, influencing both innate and adaptive immune responses [4].

Respiratory infections are a leading cause of morbidity and mortality among children and adolescents worldwide, contributing significantly to the healthcare burden [5,6]. Young children are particularly susceptible due to their developing immune systems and frequent exposure to pathogens in communal settings [7]. There is a growing interest in identifying modifiable risk factors and preventive strategies to reduce the incidence of these infections.

Several observational studies have reported an inverse association between vitamin D status and the risk of respiratory infections [8,9]. Vitamin D is thought to enhance immune function by inducing the production of antimicrobial peptides, such as cathelicidin and defensins, which can inhibit the replication of respiratory pathogens [10]. Moreover, vitamin D may modulate inflammatory responses, potentially reducing the severity of infections [11].

Despite the biological plausibility and supportive observational data, randomized controlled trials investigating the effect of vitamin D supplementation on respiratory infections have yielded inconsistent results [12,13]. Factors contributing to these discrepancies may include variations in study populations, baseline vitamin D statuses, dosing regimens, and adherence to supplementation protocols [14].

Given the high prevalence of vitamin D deficiency among children and adolescents, particularly in regions with limited sunlight exposure, understanding the relationship between vitamin D and respiratory infections is of public health importance [15]. Clarifying this association could inform guidelines on vitamin D supplementation as a preventive measure in the pediatric population.

This study aims to evaluate the association between different concentrations of vitamin D supplementation, serum 25-hydroxyvitamin D (25-OHD) concentrations, and the frequency of respiratory infections among children and adolescents aged 1 to 18 years. By stratifying participants based on supplementation doses, serum concentrations, and age groups, we seek to identify potential dose–response relationships and age-specific effects. Additionally, we perform subgroup analyses to explore other factors that may influence this association.

## 2. Materials and Methods

### 2.1. Study Design and Setting

This study was conducted as a cross-sectional investigation from January 2022 to January 2024, within the Pediatric Department of the County Clinical Hospital, a major tertiary-care facility in Timisoara, Romania. This department is associated with the Victor Babes University of Medicine and Pharmacy, also located in Timisoara. The study’s design and execution received approval from the Institutional Review Board, ensuring compliance with ethical standards as stipulated in the Declaration of Helsinki.

The participant group comprised 194 children and adolescents, ranging from 1 to 18 years of age. These participants were recruited through routine outpatient visits and at community health camps, settings that likely facilitated diverse sampling. To adhere to ethical research practices, written informed consent was secured from either the parents or legal guardians of all participants involved in the study. This careful attention to consent and ethical compliance underscores the study’s commitment to maintaining high ethical standards throughout its duration.

### 2.2. Inclusion and Exclusion Criteria

The study included children and adolescents aged between 1 and 18 years. Participants were required to have available data regarding their vitamin D supplementation and corresponding serum 25-hydroxyvitamin D (25-OHD) concentrations. Additionally, they had no known history of chronic illnesses that could influence immune system functioning or disrupt normal vitamin D metabolism.

Exclusion criteria for this study encompassed several chronic conditions and other factors. Individuals with chronic respiratory diseases, such as asthma or cystic fibrosis, were excluded. Similarly, those with chronic renal or hepatic disorders were not eligible for participation. The use of medications known to affect vitamin D metabolism, such as anticonvulsants or glucocorticoids, also disqualified potential participants. Furthermore, any recent acute illness or infection occurring within the past two weeks led to exclusion. Lastly, individuals with incomplete data or who refused to provide consent were not included in the study.

### 2.3. Laboratory Analysis

Blood samples were collected to measure serum 25-OHD concentrations using a standardized chemiluminescent immunoassay method (e.g., Roche Elecsys Vitamin D total assay). Additional laboratory tests included complete blood count (CBC), C-reactive protein (CRP), and erythrocyte sedimentation rate (ESR) to rule out active infections or inflammation at the time of sampling.

Serum 25-hydroxyvitamin D (25-OHD) concentrations were measured using the Roche Elecsys Vitamin D total assay, a chemiluminescent immunoassay method. This assay is standardized according to the manufacturer’s guidelines, which ensures reproducibility and accuracy across different laboratories and time points. We specifically adhered to the protocol recommended by Roche, which is validated by extensive inter-laboratory studies to provide a precise quantification of 25-OHD levels.

The performance of the assay was rigorously monitored by assessing both intra- and inter-assay coefficients of variation. The intra-assay coefficient of variation was maintained below 5%, ensuring consistent results within individual assay batches. The inter-assay coefficient of variation was kept under 10%, affirming reliability across multiple assay runs conducted over the duration of the study.

All serum assays were performed in a single batch at the end of the collection period to avoid variability associated with time-dependent factors such as reagent stability and calibration drift. This approach minimized potential biases that could have arisen from analyzing samples at different times, particularly given the cross-sectional nature of our study and the diverse age range of the participants.

### 2.4. Data Collection and Definitions

Data for this study were gathered using structured questionnaires and medical records, capturing a range of information including demographic data (age, gender), anthropometric measurements (height, weight, BMI), details on vitamin D supplementation (dosage and frequency), dietary intake, sun exposure habits, sunscreen use, and history of respiratory infections over the past 12 months. The study defined a respiratory infection as an episode with symptoms such as cough, sore throat, runny nose, fever, or difficulty breathing that was diagnosed by a healthcare professional. Recurrent respiratory infections were classified as two or more episodes within the past 12 months. Vitamin D supplementation was categorized into three concentrations: low (less than 400 IU per week), moderate (400–800 IU per week), and high (more than 800 IU per week). Serum 25-OHD concentrations were classified as deficient (less than 20 ng/mL), insufficient (20–30 ng/mL), and sufficient (more than 30 ng/mL). These classification thresholds were derived from the guidelines provided by the Endocrine Society, which are widely recognized for the clinical assessment of vitamin D status [16].

In this cross-sectional study, we gathered self-reported data on vitamin D supplementation, noting the variety of pharmacological forms used by participants. The most common formulations of supplemental vitamin D for children, as reported in our study, included cholecalciferol (vitamin D3) and ergocalciferol (vitamin D2), with cholecalciferol being more prevalent due to its higher efficacy in raising serum 25-hydroxyvitamin D levels. These were administered in forms such as oral drops, which are often favored for ease of dosing in young children, and high-potency softgel capsules, which are suitable for older children. The study did not specify or provide these supplements; instead, it recorded detailed information on the type, dosage, and frequency of vitamin D intake as reported by the participants or their guardians.

### 2.5. Statistical Analysis

Statistical analyses in the study were conducted using IBM SPSS Statistics, Version 26.0 (Armonk, NY, USA). We reported continuous variables using either the mean with standard deviation (SD) or the median with interquartile range (IQR), depending on the data’s distribution. For categorical data, we presented frequencies and percentages. To compare data across various groups, we employed chi-square tests for categorical variables and either one-way ANOVA or Kruskal–Wallis tests for continuous variables, depending on the normality of the data distribution. We also carried out post hoc comparisons using the Bonferroni correction method to adjust for multiple testing effects. Additionally, logistic regression models were utilized to explore the relationships between vitamin D supplementation, serum vitamin D levels, and the frequency of respiratory infections, while controlling for potential confounding factors such as age, gender, body mass index (BMI), sun exposure habits, and sunscreen usage. Statistical significance was established at a *p*-value of less than 0.05, using a two-tailed test. This comprehensive approach allowed us to robustly assess the impact of vitamin D on health outcomes while accounting for various influencing factors.

## 3. Results

### Background Characteristics

Significant differences were observed in the mean age and BMI across the three age groups, with the youngest group (<6 years) having the lowest mean age and BMI and the oldest group (12–18 years) having the highest (*p* < 0.001 for both age and BMI). The proportion of males was relatively consistent across all age groups, with about half of the participants in each group being male, resulting in no significant difference in gender distribution (*p* = 0.987). Although there was an increase in the proportion of overweight or obese participants with age, this trend was not statistically significant (*p* = 0.182). Similarly, differences in sun exposure and daily sunscreen use among the groups were not significant (*p* = 0.301 and *p* = 9.920, respectively), suggesting similar lifestyle behaviors regarding sun-related activities across different age groups (Table 1).

The results demonstrated a clear positive correlation between higher vitamin D supplementation concentrations and increased serum 25-OHD concentrations in all age groups. Specifically, for children under 6 years, those receiving over 800 IU/week exhibited the highest mean serum 25-OHD concentration at 33.0 ± 6.2 ng/mL, significantly higher compared to those receiving less than 400 IU/week, who had a mean concentration of 18.2 ± 4.5 ng/mL (*p* = 0.0041). Similarly, in the 6-12 years group, children receiving over 800 IU/week showed a significant increase in serum 25-OHD concentrations (31.5 ± 5.8 ng/mL) compared to those receiving less than 400 IU/week (17.5 ± 3.8 ng/mL) with a *p*-value of <0.001. Adolescents aged 12–18 years followed this trend, where those supplemented with more than 800 IU/week had mean serum concentrations of 29.8 ± 5.5 ng/mL, significantly higher than those receiving less than 400 IU/week (16.8 ± 3.5 ng/mL), also with a *p*-value of <0.001. This pattern suggests that higher doses of vitamin D supplementation are effectively associated with higher circulating concentrations of 25-OHD across all pediatric and adolescent age groups (Table 2).

A statistically significant association was observed between vitamin D supplementation concentrations and the incidence of respiratory infections (*p* < 0.001). The incidence of infections decreased as the concentration of vitamin D supplementation increased. Specifically, participants receiving less than 400 IU/week experienced the highest incidence rate at 60.00%, those receiving between 400 and 800 IU/week had a lower rate of 43.80%, and the group supplemented with more than 800 IU/week showed the lowest incidence rate at only 16.70%, as presented in Table 3.

As serum 25-OHD concentrations increased with higher supplementation, the incidence of having two or more respiratory infections notably decreased. Specifically, the group receiving less than 400 IU/week of vitamin D had the highest incidence rate at 61.4%. In contrast, those receiving between 400 and 800 IU/week saw a reduced incidence of 42.2%, and the group with the highest supplementation (>800 IU/week) had the lowest incidence at only 16.7%, as seen in Table 4.

**Figure 1:**
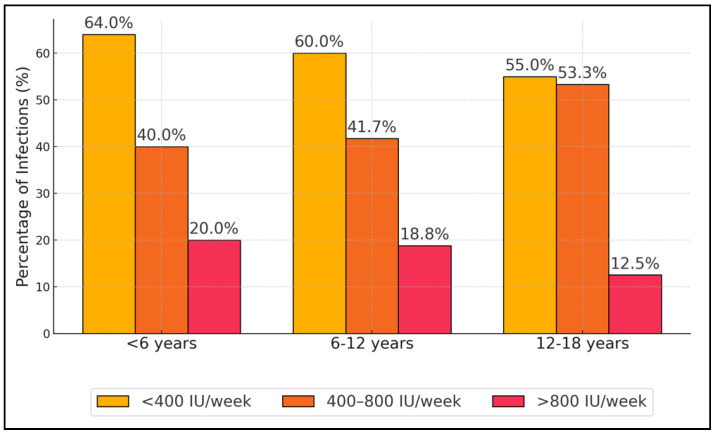
Incidence of respiratory infections by vitamin D supplementation level in different age groups.

In the youngest group (<6 years), those receiving less than 400 IU/week of vitamin D had the highest incidence rate at 64.00%. This rate decreased to 40.00% in those receiving between 400 and 800 IU/week and further dropped to 20.00% among those receiving more than 800 IU/week, with a total group *p*-value of 0.002. A similar trend was observed in the 6–12 years age group, where the incidence rates were 60.00% for less than 400 IU/week, 41.70% for 400–800 IU/week, and 18.80% for more than 800 IU/week, with a *p*-value of 0.016. The oldest age group (12–18 years) also showed a reduction in infection rates with increased vitamin D dosage: 55.00% for less than 400 IU/week, 53.30% for 400–800 IU/week, and significantly lower at 12.50% for more than 800 IU/week, with a *p*-value of 0.033 (Table 5, and Figure 1).

Vitamin D supplementation above 800 IU/week was associated with a substantial reduction in the odds of infection, with an odds ratio (OR) of 0.28 and a 95% confidence interval (CI) of 0.12–0.52 (*p* < 0.001). Similarly, having serum 25-OHD concentrations above 30 ng/mL also significantly reduced the odds of infections, with an OR of 0.23 and a 95% CI of 0.10–0.49 (*p* < 0.001). Conversely, other variables such as age group, BMI category, sun exposure, and daily sunscreen use did not show significant associations with the incidence of respiratory infections. The odds ratios for age groups 6–12 years and 12–18 years were 1.05 (95% CI: 0.63–1.84, *p* = 0.866) and 1.08 (95% CI: 0.55–1.83, *p* = 0.915), respectively, indicating no significant difference in infection risk compared to the reference group (<6 years). BMI categorized as overweight/obese, sun exposure greater than 30 min per day, and daily sunscreen use also did not show significant relationships with respiratory infection risks, with *p*-values well above 0.05, as presented in Table 6.

## 4. Discussion

### 4.1. Literature Findings

The current findings suggest that higher vitamin D supplementation (>800 IU/week) and sufficient serum 25-OHD concentrations (>30 ng/mL) are significantly associated with a lower incidence of respiratory infections. The overall incidence of respiratory infections in the study population was 41.2%, consistent with reported rates in similar age groups [17]. Participants receiving higher concentrations of vitamin D supplementation had significantly lower infection rates. Specifically, only 16.7% of participants receiving >800 IU/week experienced recurrent respiratory infections, compared to 60.0% in the <400 IU/week group. This dose–response relationship supports the hypothesis that adequate vitamin D supplementation may enhance immune defense mechanisms.

Similarly, participants with sufficient serum 25-OHD concentrations had a markedly lower incidence of infections. The infection rate was 16.7% in those with serum concentrations >30 ng/mL, compared to 61.4% in those with concentrations <20 ng/mL. These findings are in line with previous studies demonstrating an inverse relationship between vitamin D status and respiratory infection risk [18,19,20].

Subgroup analyses revealed that the protective effect of vitamin D was significant across all age groups but was most pronounced in children under 6 years old. This age group is particularly vulnerable to respiratory infections due to immature immune systems and high exposure in communal settings such as daycare and preschool [21]. The significant reduction in infection rates among younger children suggests that early intervention with vitamin D supplementation may be especially beneficial.

The logistic regression analysis confirmed that higher vitamin D supplementation and sufficient serum 25-OHD concentrations were independently associated with reduced odds of respiratory infections, even after adjusting for potential confounders. Other factors such as age, gender, BMI, sun exposure, and sunscreen use did not show significant associations, indicating that vitamin D status plays a more critical role. The mechanisms by which vitamin D may reduce respiratory infections include the induction of antimicrobial peptides, the modulation of cytokine production, and the enhancement of mucosal immunity [22,23]. Vitamin D receptors are expressed in various immune cells, and vitamin D can influence the function of macrophages, dendritic cells, and T cells, promoting an effective immune response against pathogens [24,25].

In a similar manner, the study by Jaybhaye et al. [26] highlighted a strong correlation between suboptimal vitamin D concentrations and recurrent respiratory infections in children, where 25% were deficient and 75% were insufficient in vitamin D among the cases. This significant association (*p* < 0.001) underscores the high prevalence of vitamin D deficiency among children with recurrent respiratory infections compared to controls, advocating for the inclusion of vitamin D status assessment in their management. Conversely, the research conducted by Yakoob et al. [27] investigated the preventive role of vitamin D supplementation against infections like pneumonia and diarrhea in children under five years, but the findings were more inconclusive. Despite the trials indicating a higher mean serum vitamin D concentration in supplemented children, the outcomes such as pneumonia incidence and hospitalization showed no significant differences between the supplemented and unsupplemented groups. Particularly, in Afghanistan, vitamin D supplementation was linked with an increase in repeat pneumonia episodes, contrary to expectations. This paradoxical result from a large trial (RR 1.69, CI 1.28 to 2.21) points to the complex interplay between vitamin D supplementation and respiratory health outcomes in diverse geographical settings, suggesting that vitamin D may not uniformly prevent infections across different child populations.

In the systematic review and meta-analysis conducted by Jolliffe et al. [28], vitamin D supplementation demonstrated a modest but statistically significant reduction in the risk of acute respiratory infections (ARIs) compared to a placebo, with an overall odds ratio (OR) of 0.91, indicating a 9% reduction in risk. The study underscored that daily dosing regimens were particularly effective, displaying protective effects with ORs ranging from 0.70 to 0.75 for daily doses of 400–1000 IU and for durations up to 12 months. This finding emphasizes the potential benefits of consistent, moderate-dose vitamin D supplementation over time. In a similar manner, the study by Loeb et al. [29] on a population of Vietnamese children and adolescents did not observe a significant reduction in influenza infections with high-dose weekly vitamin D supplementation (14,000 IU), as evidenced by a hazard ratio (HR) of 1.18. However, it did find a moderate reduction in non-influenza respiratory virus infections with an HR of 0.76, reinforcing the notion that vitamin D’s protective effects might be more pronounced against certain types of respiratory pathogens.

Similarly, in the study by Reyes et al. [30], weekly vitamin D supplementation was assessed for its effectiveness in reducing acute respiratory infections (ARIs) in preschool children across different latitudes in Chile. Despite a significant dose-dependent increase in serum 25-hydroxyvitamin D (25(OH)D) concentrations in children receiving vitamin D3 supplements (5600 IU/week and 11,200 IU/week), the trial reported no significant reduction in the number of ARIs, ARI hospitalizations, changes in LL-37/cathelicidin concentrations, or adverse events, indicating that higher internal vitamin D concentrations did not correlate with fewer respiratory infections. Similarly, the study by Umeadi et al. [31] explored the association between serum vitamin D concentrations and the incidence of ARIs in under-five children in Nigeria, revealing that children with acute lower respiratory infection (ALRI) had significantly lower mean serum 25(OH)D concentrations compared to those with upper respiratory infections (AURIs) and controls. This finding suggests a potential link between lower vitamin D concentrations and the severity of respiratory infections, particularly ALRIs.

The omission of dietary vitamin D as a continuous variable was primarily due to the challenges associated with accurately measuring and quantifying dietary intake in a cross-sectional study format. Typically, dietary assessments require detailed food frequency questionnaires or dietary records, which were not feasible within the scope of our study parameters. However, it is recognized that the assumption that the typical Romanian diet provides negligible vitamin D may not adequately reflect the actual dietary habits and variations among the population. This assumption was based on general dietary patterns observed in Eastern Europe, where natural food sources rich in vitamin D, such as fatty fish or fortified foods, are less commonly consumed. Nevertheless, the lack of specific dietary data could indeed confound the results, potentially attributing variations in serum vitamin D levels more to supplementation and less to diet than might be accurate.

Building on the insights provided by this manuscript, future research should aim to delineate the specific protective concentrations of vitamin D against various types of infections, particularly distinguishing between viral and bacterial pathogens. This would involve conducting more comprehensive longitudinal studies to monitor vitamin D concentrations and infection outcomes over extended periods. Additionally, interventional studies could be designed to assess the efficacy of vitamin D supplementation in enhancing immune response and reducing the incidence of pediatric infections. Such studies should employ a rigorous methodology to control for potential confounders and ensure the reliability of the results. Expanding the demographic and geographic scope of the research could also provide insights into how environmental factors and genetic predispositions interact with vitamin D to influence immune function. Ultimately, these studies could lead to more targeted public health strategies and interventions aimed at optimizing vitamin D concentrations to prevent infections in children.

### 4.2. Limitations

Despite the promising findings, the study has limitations. The cross-sectional design precludes establishing causality, and temporal relationships cannot be confirmed. Self-reported data on vitamin D supplementation and respiratory infections may be subject to recall bias. Serum 25-OHD concentrations were measured at a single time point, which may not reflect long-term vitamin D status. Additionally, residual confounding factors may exist, although we adjusted for several potential confounders.

## 5. Conclusions

This study suggests that higher vitamin D supplementation and sufficient serum 25-OHD concentrations are associated with a reduced incidence of respiratory infections among children and adolescents. The protective effect is significant across all age groups and is particularly notable in children under 6 years old. These findings support the importance of maintaining adequate vitamin D status in the pediatric population as a potential strategy to prevent respiratory infections. Public health initiatives promoting appropriate vitamin D supplementation may contribute to reducing the burden of respiratory infections in children and adolescents.

## Figures and Tables

**Table 1:** Demographic and clinical characteristics by age group.

Variable	<6 Years (*n* = 70)	6–12 Years (*n* = 65)	12–18 Years (*n* = 59)	Total (*n* = 194)	*p*-Value
Age, mean ± SD (years)	3.8 ± 1.2	9.0 ± 1.9	15.1 ± 1.8	9.6 ± 5.1	<0.001
Gender (Male), *n* (%)	36 (51.4%)	34 (52.3%)	30 (50.8%)	100 (51.5%)	0.987
BMI, mean ± SD (kg/m²)	15.9 ± 1.8	18.1 ± 2.5	21.7 ± 3.2	18.3 ± 3.6	<0.001
Overweight/Obese, *n* (%)	8 (11.4%)	12 (18.5%)	14 (23.7%)	34 (17.5%)	0.182
Sun Exposure (>30 min/day), *n* (%)	50 (71.4%)	40 (61.5%)	35 (59.3%)	125 (64.4%)	0.301
Daily Sunscreen Use, *n* (%)	20 (28.6%)	18 (27.7%)	15 (25.4%)	53 (27.3%)	9.920

SD—standard deviation; BMI—body mass index.

**Table 2:** Vitamin D supplementation and serum 25-OHD concentrations by age group.

Age Group	Vitamin D Supplementation Concentration	*n*	Mean Serum 25-OHD (ng/mL) ± SD	*p*-Value
<6 years				0.0041
	<400 IU/week	25	18.2 ± 4.5	
	400–800 IU/week	25	24.5 ± 5.0	
	>800 IU/week	20	33.0 ± 6.2	
6–12 years				<0.001
	<400 IU/week	25	17.5 ± 3.8	
	400–800 IU/week	24	23.8 ± 4.6	
	>800 IU/week	16	31.5 ± 5.8	
12–18 years				<0.001
	<400 IU/week	20	16.8 ± 3.5	
	400–800 IU/week	15	22.5 ± 4.2	
	>800 IU/week	24	29.8 ± 5.5	

SD—standard deviation; IU—international units; 25-OHD—25-hydroxyvitamin D_3_.

**Table 3:** Incidence of respiratory infections by vitamin D supplementation concentrations.

Variables (Mean ± SD)	1 Time (*n* = 74)	2–3 Times (*n* = 89)	>3 Times (*n* = 52)	*p*-Value
<400 IU/week	70	42	60.00%	
400–800 IU/week	64	28	43.80%	
>800 IU/week	60	10	16.70%	
Total	194	80	41.20%	<0.001

SD—standard deviation; IU—international units.

**Table 4:** Incidence of respiratory infections by serum 25-OHD concentrations.

Serum 25-OHD (ng/mL) ± SD	*n*	Number with ≥2 Infections	Percentage (%)	*p*-Value
<400 IU/week	70	43	61.4%	
400–800 IU/week	64	27	42.2%	
>800 IU/week	60	10	16.7%	
Total	194	80	41.2%	<0.001

SD—standard deviation; IU—international units.

**Table 5:** Incidence of respiratory infections by vitamin D supplementation in different age groups.

Age Group	Serum 25-OHD (ng/mL) ± SD	*n*	Number with ≥2 Infections	Percentage (%)	*p*-Value
<6 years	<400 IU/week	25	16	64.00%	
	400–800 IU/week	25	10	40.00%	
	>800 IU/week	20	4	20.00%	
Total		70	30	42.90%	0.002
6–12 years	<400 IU/week	25	15	60.00%	
	400–800 IU/week	24	10	41.70%	
	>800 IU/week	16	3	18.80%	
Total		65	28	43.10%	0.016
12–18 years	<400 IU/week	20	11	55.00%	
	400–800 IU/week	15	8	53.30%	
	>800 IU/week	24	3	12.50%	
Total		59	22	37.30%	0.033

SD—standard deviation; IU—international units; 25-OHD—25-hydroxyvitamin D_3_.

**Table 6:** Logistic regression analysis of factors associated with respiratory infections.

**Variable**	**Odds Ratio (OR)**	**95% Confidence Interval (CI)**	***p*** **-Value**
Vitamin D Supplementation (>800 IU/week)	0.28	0.12–0.52	<0.001
Serum 25-OHD (>30 ng/mL)	0.23	0.10–0.49	<0.001
Age Group (6–12 years)	1.05	0.63–1.84	0.866
Age Group (12–18 years)	1.08	0.55–1.83	0.915
BMI (Overweight/Obese)	1.21	0.68–2.23	0.562
Sun Exposure (>30 min/day)	0.94	0.50–1.62	0.741
Daily Sunscreen Use	1.19	0.61–2.07	0.763

BMI—body mass index; IU—international units; 25-OHD—25-hydroxyvitamin D_3_.

## Data Availability

Data supporting reported results can be obtained from the corresponding author. Data are not publicly available due to privacy and ethical reasons.
